# Elevation-driven variations in soil-plant stoichiometry and heavy metal dynamics in coniferous and Oak Forests of Gilgit Baltistan (GB), Pakistan

**DOI:** 10.1371/journal.pone.0360614

**Published:** 2026-10-26

**Authors:** Ahmad Jan, Muhammad Arshad, Muhammad Tayyab Khan, Shazia Khushdil, Fariha Rehman, Majid Hussain, Ashfaq Ali, Tufail Rahman, Faisal Khalid

**Affiliations:** 1 Department of Forestry, Range & Wildlife Management, Karakoram International University, Gilgit-Baltistan, Pakistan; 2 Land Resources Research institute-NARC, PARC, Islamabad, Pakistan; 3 Department of Land, Environment, Agriculture and Forestry, University of Padua, Via Università, Legnaro, Padua, Italy; 4 Department of Forestry, Shaheed Benazir Bhutto University, Khyber Pakhtunkhwa, Pakistan; 5 Department of Economics, COMSATS University Islamabad (CUI), Lahore Campus, Lahore, Punjab, Pakistan; 6 Department of Forestry & Wildlife Management, The University of Haripur, Khyber Pakhtunkhwa, Pakistan; 7 GIS and Space Applications in Geosciences Lab (GSAG-L), National Center of GIS and Space Application (NCGSA), Institute of Space Technology, Islamabad, Punjab, Pakistan; Bangor University, UNITED KINGDOM OF GREAT BRITAIN AND NORTHERN IRELAND

## Abstract

This study examined the effects of elevation gradients on on soil and plant stoichiometry and heavy metals distribution in Mixed Coniferous Forests (MCF) and Oak Forests (OF). Soil organic carbon (SOC), total nitrogen (TN), and total phosphorus (TP) were significantly higher at the 10 cm soil depth at elevation EL-2, followed by EL-3, in both forests; whereas the lowest values were observed at EL-1. MCF exhibited significantly higher SOC, TN, and TP concentrations than OF. Similarly, leaf carbon, nitrogen, and phosphorus concentrations were highest EL-2. In oak forests, leaf carbon, nitrogen, and phosphorus concentrations reached 458.4 mg kg ^−^ ¹, 4.42 mg kg ^−^ ¹, and 2.54 mg kg ^−^ ¹, respectively. The Corresponding values in MCF were 472 mg kg ^−^ ¹, 16.1 mg kg ^−^ ¹, and 1.9 mg kg ^−^ ¹, respectively, indicating greater nutrient accumulation at the mid-elevation site (EL-2). The leaf C:N ratio was significantly higher at EL-2, whereas the C:P and N:P ratios did not differ significantly among elevations. A similar pattern was observed in branches, bark, and litter. At EL-2 site, soil nickel (Ni), lead (Pb), cadmium (Cd), copper (Cu), and iron (Fe) concentrations at the 10 cm soil depth were significantly lower in MCF than in OF. Conversely, plant tissues in MCF exhibited higher concentrations of Ni, Pb, zinc (Zn), Cd, Cu, and Fe than those in OF at EL-1. Soil and plant stoichiometry are fundamental components of ecosystem functioning and nutrient cycling, and elevation gradients also play a critical role in regulating these processes. These findings offer valuable insights for developing sustainable forest management strategies that aim to mitigate heavy metal pollution in high-altitude ecosystems.

## 1. Introduction

The balance of energy and multiple chemical elements in ecological interactions, a concept known as ecological stoichiometry (ES), provides a useful perspective for understanding the biogeochemical cycling of terrestrial ecosystems [1]. The ratios of the three important biological elements—C, N, and P—in soils and plants are not merely passive outcomes but fundamental characteristics that regulate physiological processes, resource-use efficiency, and decomposition rates [2]. C:N:P stoichiometry is an important indicator for evaluating the responses and adaptation of an organism to environmental changes, reflecting nutrient limitation and environmental conditions [3, 4]. As a result, the large-scale distribution of foliar and soil concentrations and concentration ratios of elements has been widely investigated from local to global scales, revealing large-scale biogeographical patterns [5,6], and ecosystem responses to global change. But the complex balance of these nutrient cycles can be disrupted by pollutants. Heavy metal contamination of soil, driven by industrial activity, mining, and agricultural intensification, significantly threatens these fragile biochemical equilibria [7,8]. The toxic effects of metals such as cadmium (Cd), lead (Pb), copper (Cu) or zinc (Zn) are associated with nutrient competition, due to interference with nutrient absorption and utilization, disruption of cellular nutrient metabolism, thereby altering plant C:N:P stoichiometry, lower growth rates and fitness.

This degree of disturbance is, however, not homogeneous but rather largely controlled by soil properties (e.g., pH, organic matter content) as well as plant species traits that are inherent in both tolerance- and exclusion-strategies. In the terrestrial biosphere, forests are considered critical components contributing to global nutrient cycling, carbon storage, and biodiversity [9]. The stoichiometry of forest soils and vegetation is a major driver of these ecosystem services. Forest ecosystems are dynamic, particularly during secondary succession, and their stoichiometry is therefore not spatially or temporally uniform. Forest ecosystems are dynamic, which is particularly true in secondary growth stands, and thus not statically uniform. For example, C:N ratios in plant tissues and soils can change substantially across stand-development stages due to age-mediated competition, light availability, and changes in nutrient allocation [10,11]. Although some studies have found that C:N ratios decrease with stand age, other research shows the relationship of C: N: P to be complex, non-linear, and not fully understood [12]. This raises an important knowledge gap: the requirement to combine spatial and temporal information and predict responses of forest stoichiometry to co-varying environmental drivers. A key predictor of ecosystem stoichiometry is the kind of forest cover, that is, whether coniferous or broadleaved stands will prevail.

Broadleaved species, such as an oak forest) tend to contain higher nutrient concentrations (N and P) and exhibit faster nutrient turnover than coniferous forests. In contrast, coniferous forests, including mixed coniferous forests (MCF), generally produce litter with higher C:N ratios and slower decomposition rates [13,14]. These differences have direct effects on the soil. Plant functional type differences impact soil. Broadleaved litterfall mineralizes more rapidly and returns nutrients to the soil, whereas conifer litter has been cited as causing a build-up of an organic acidified layer in the soil [15]. This, in turn, created separate soil microhabitats that may all be able to affect the fate and bioavailability of heavy metals differently. One of these hypotheses is the existence of a general pattern of eco-physiological differences among forest types determined by different patterns in nutrient relations, which are developed by those forests. Mountain landscapes provide natural laboratories for understanding how environmental gradients influence ecosystem processes. A mountain landscape is an outstanding natural laboratory for us to understand how the climatic factors affect processes in the soil as steep elevational gradients produce pronounced climatic changes over relatively short horizontal distances (e.g., temperature and rainfall) at short distances and lead to significant shifts in vegetation, microbial activity and soil processes [16–18]. Colder temperatures with higher elevation slow the degradation of litter and add to the SOC stock and thickness of the organic horizon [19], resulting in changes in nutrient cycling through exacerbated nitrogen limitation [20]. These elevation-controlled shifts have far-reaching effects on heavy metal pollution because there are significant changes in soil chemistry, hydrology, and metal mobility along elevation gradients [21,22]. Soil properties mediate the eventual fate of metals, such as high SOC potentially providing a metal sink [23], whereas soil acidity can increase metal mobility and bioavailability [24]. These contrasting nutrient dynamics suggest that forest type plays an important role in regulating ecosystem stoichiometry and heavy metal cycling.

Forest canopies also intercept deposition and funnel metals through throughfall, stemflow, and litterfall [25,26], although plant uptake is species-dependent [27] and bioaccumulation is significant even in pristine high mountain ecosystems [28]. Despite considerable research on soil–plant interactions and heavy metal contamination, an integrated understanding of how elevation simultaneously influences soil–plant stoichiometry and heavy metal accumulation remains lacking. Knowledge Gap and Problem Statement. However, although there are abundant reports of soil-plant interactions and various studies on heavy metal contamination, a wide knowledge gap and consistency have not been reached; we still lack an integrated understanding of how elevation influences the mutual relationship between soil-plant stoichiometry (C: N: P) and heavy metal accumulation [29]. Earlier studies treated these elements in isolation. While elevation affects nutrient and metal distribution, we lack consensus on how high-altitude processes interact to shape ecosystem health. Altitude also alters microclimate, soil properties, and plant communities, driving changes in elemental stoichiometry and metal biogeochemical cycling [30,31]. Research is therefore required on the C:N:P ratios of soil and plants at various elevations, as well as any possible connections between these two ratios’ variation patterns and heavy metals like Pb, Cd, Zn, and Cu in terms of their spatial distributions, bioavailability, and translocations [32].

Moreover, the potential risk of these metals to the environment might not be homogeneous at different elevation levels, and a tailored evaluation should be employed based on standard predictive indices (e.g.[12],. It is especially a yawning gap in the world’s great mountain ranges, the proverbial canaries in the planetary coal mine. The present study was conducted in the Pakistani sectors of the Hindu Kush, Karakoram, and Himalayan mountain ranges—regions that remain underexplored in the context of ecological stoichiometry despite their ecological significance and heightened vulnerability to anthropogenic stress. By combining ecological stoichiometry with heavy-metal biogeochemistry, this work provides new insights into forest ecosystem functioning along elevational gradients. The study providescrucial baseline information for understanding how soil–plant interactions shift with elevation, with direct implications for conservation planning and the sustainable management of these ecologically sensitive mountain systems. The findings alsocontributes to environmental theory by addressing existing gaps in knowledge regarding nutrient–metal dynamics along ecological gradients. From an applied perspective, the findings provide quantitative evidence that can support policymakers seeking to mitigate pollution in high-altitude regions that function as essential water towers for downstream populations. Specifically, this study investigates changes in soil and plant C, N, and P stoichiometry across elevations in mixed coniferous and oak forests; evaluates the concentrations, bioavailability, and translocation of heavy metals from soil to plants across elevational zones; and assesses associated environmental risks using established ecological risk indices.

## 2. Materials and methods

### 2.1. Study area

This study was conducted in Tangir Valley of Gilgit-Baltistan, in the northern mountains of Pakistan. The study area is located at 35.63° N latitude and 73.45° E longitudeat an elevation of 1767.77 m a.s.l. The study area lies closer to some of the highest peaks of the Himalayan and Karakoram mountain ranges. Due to its diverse climatic conditions, the area has rich biodiversity with pine forests, oak forests, and alpine meadows. These forests are home to a diverse wildlife such as the Markhor (*Capra falconeri*), Himalayan ibex (*Capra ibex*), Snow Leopards (*Panthera uncia),* and a variety of bird species. Within the major forests of the area several sub-forests also exist such as the Mixed Coniferous Forests (MCF), Pure Oak Forests (OF), and Subalpine forests. Brown mountainous soil constitutes the main soil type. Summers are generally less warmer while winters are cold with significant snowfall Mean annual temperature ranges from 11–15°C in the lower elevations with a maximum of 35°C in July. Winters are commonly cold and dry with a temperature of 0°C while it remains below 0°C in December to February. The valley receives a mean annual precipitation (MAP) of 300–500 mm in valley floor while the high-altitude peaks receive significantly higher amount of moisture during winters.

### 2.2. Sampling design

In the current study we adopted the elevation gradient strategy to test our objectives. Four elevation classes EL-1 (1000-1400m), EL-2 (1501-1900m), EL-3 (2001-2300m), and EL-4 (>2401m). We collected data from each elevation class using cluster sampling (random sampling) method. This sampling method consists of the Primary Sample Plots (PSUs) and Secondary Sample Plots (SSUs). In this study, cluster (random) sampling including composite methodology, was employed (Bried and [33]. Every cluster was given a distinct number/reference/code/ID (pre-indexed). The number of clusters was produced in GIS. From each cluster, plots (PSUs and SSUs) were assigned numbers1–5. Number 5 was allocated to each cluster as the Primary Sampling Unit (PSU). The bottom-left SSU was assigned label 1, then one clockwise as 2, 3, and the top-rightmost was numbered 4. Plot ID was generated fromcluster number + plot number and was generated automatically in GIS/database. Plot IDs were generated in GIS using:


$$\begin{array}{c} \text{Plot ID}=\text{Cluster Number }+\text{ Plot Number} \end{array}$$

### 2.3. Measurements

In the study are the germinating period ranges from March to mid-October, so during this period significant vegetation growth is obvious. Therefore, sampling was conducted during July and early October 2024 in order to collect samples of both green and senesced leaves. Fresh green leaves were collected by hand from the mid-canopy branches of each 5-standard tree/plot to ensure representativeness of the samples. Soil samples were taken at three depths, e.g., topsoil (0–10 cm), middle soil layer (10–20 cm), and subsoil layer (20–30 cm). In the study area soil samples were collected from three cluster random sampling points with a soil augerto a depth of 30 cm. These samples were pooled at their respective depths to obtain one bulk soil sample and one disturbed soil sample per depth for each plot. We collected a total of 36 soil samples [(3 × 3 × 4) = 36], with the same number of compact soil and loose soil from three clusters. We also collected leaves, twigs, bark, and litter from each plot to provide a full data set for our experiment. A total of 36 soil samples were collected at each elevation with 12 leaf samples for each category (Green and Senesced).


$$\begin{array}{c} \text{Total Soil Samples in each elevation }(\text{EL})=\text{3}\text{ clusters}\times \text{3}\text{ depths}\times \text{4}\text{ plots}=\text{36} \end{array}$$

In order to standardize drying treatments, samples were initially subjected to 105°C for 15 min before being further dried at 85°C until a constant mass was determined. Then, we performed a Focusing (TC, TN, and TP) in these dried samples [34,35]. The analysis of TC and TN concentrations was also carried out on soil samples.

### 2.4. Lab investigation

Upon arrival in the laboratory, soil samples from MCF and Oak Forest were air-dried, sieved to remove alien materials and coarser fractions. The carbon content in rooted samples is determined by the elemental analysis or using the Walkley-Black method for soil organic carbon determination [36].


$$\begin{array}{c} \text{SOC }(\%)=\frac{(V_{\text{sample}}-V_{balnk})\times \text{N}\times \text{0}.\text{003}\times \text{100}}{Weightofsoil(g)} \end{array}$$

where V_sample_ and V_blank_ are titration volumes (mL) and Nis the normality of ferrous ammonium sulfate. Analysis of Total Nitrogen was conducted using the Kjeldahl or elemental analyzer method, which involves oxidizing a sample and measuring the released nitrogen gas. For phosphorus, digestible samples were digested with an acid‐extraction method, such as the Olsen or Bray method, and analyzed calorimetrically in a spectrophotometer [37]. Stoichiometric ratios were calculated as mass ratios:


$$\begin{array}{c} C:N=\frac{\text{TC}}{TN},C:P=\frac{\text{TC}}{TP}\;,N:P=\frac{\text{TN}}{TP} \end{array}$$

Regression analysis was conducted to examine relationships between soil and plant CNP:


$$\begin{array}{c} Y=\beta_{\text{0}}+\beta_{\text{1}}X+\varepsilon \end{array}$$

where Y represents plant C, N, or P, X is soil C, N, or P, β_0_ is the intercept, β_1_ is the regression coefficient, and ϵ is the error term. Pearson correlation coefficients were calculated as:


$$\begin{array}{c} \text{r}=\frac{\sum ({\text{X}}_{\text{i}}-\overset{―}{\text{X}}({\text{Y}}_{\text{i}}-\overset{―}{\text{Y}})}{\sqrt{\sum {\left({\text{X}}_{\text{i}}-\overset{―}{\text{X}}\right)}^{\text{2}}}\;\sqrt{\sum {\left({\text{Y}}_{\text{i}}-\overset{―}{\text{Y}}\right)}^{\text{2}}}} \end{array}$$

r is the Pearson correlation coefficient; Xi and Yi are observations of two matched variables; X- and Y- are the means; and n is the sample size. This ratio was used to measure the levels of association between forest cover and fragmentation indices, and urban expansion and MSPA structural classes. The data generated from these analyses were consequently subjected to statistical analysis for the comparison of nutrient contents between different sampling sites or soil depths. We also assessed TC, TN, and TP in these soil, leaf, branches, bark, and litter of MCF- and Oak- plants samples to gain on C: N; C: P and N: P ratios. The stoichiometries are presented as mass ratios. The regularity/divergence of homogeneity of the data was meticulously checked, and, when applicable, they had to be rebuilt. To explore how the contents of (CNP) were associated, a regression analysis between CNP and MCF vs Oak plants and soil to make comparisons among the different soil-plant combinations was performed. The Pearson correlation analysis was conducted to determine the connection between soil C, N, P contents, Plant C, N, P contents, their ratios, and soil and plant heavy metal contents. For a broad understanding of the connection between soil stoichiometry and soil features, Principal component analysis (PCA) was employed to uncover and elucidate these relationships within the dataset.

### 2.5. Statistical analysis

Before statistics analysis, we evaluated the regularity and alteration of homogeneity for all parameters; when necessary, fact transformations were applied. A one-way analysis of variance (ANOVA) was conducted to examine the effect of elevational gradient classes, followed by an LSD test to identify significant differences among these groups with a significance threshold set at 0.05. Regression analysis was employed to explore relationships between soil and MCF and Oak plant (CNP), as well as between soil and both types of leaves. Data analyses were carried out using RStudio software to investigate the linear relationships among soil and plant stoichiometry. We performed principal component analysis (PCA) and structural equation modeling to examine the relationships between soil and plant CNP and heavy metals at different elevational gradients using RStudio software.


$$\begin{array}{c} F=\frac{{MS}_{\text{between}}}{{MS}_{within}} \end{array}$$

where MS_between_ and MS_within_ are the mean squares between and within groups. Statistical significance was set at *p* < 0.05p < 0.05p < 0.05, and analyses were conducted in RStudio.

There is no human and animal experimentation and investigation in this study therefore no requirement of ethical approval from Ethical committee of the University was required.

## 3. Results

### 3.1. Variations in soil stoichiometry between Mixed coniferous and Oak forests at different elevations (EL)

The results of soil nutrients concentrations of MCF and OF forests at different soil depths for different elevations are shown in Fig 1. Results showed that soil organic carbon (SOC) was significantly higher in the MCF compared with that of the Oak forest at the 0–10 cm soil depth at elevation EL-2. The Oak Forest reported higher SOC contents, such as 8.6 g kg^−1^, 7.6 g kg^−1^, 6.2 g kg^−1^, and 5.1 g kg^−1^ at EL-2, followed by EL-3, EL-1, and EL-4 at the 0–10 cm soil depth. The SOC content decreased with the increase in depth. A similar trend was noted in the MCF that SOC content was significantly improved by 54 g kg^−1^ (EL-2), 46 g kg^−1^ (EL-3), 38 g kg^−1^ (EL-1), and 35 g kg^−1^ (EL-4) at a 10 cm depth of soil. Moreover, the comparison results of SOC between MCF and OF showed that the maximum soil organic carbon was noted in MCF compared with that of OF.

**Fig 1:**
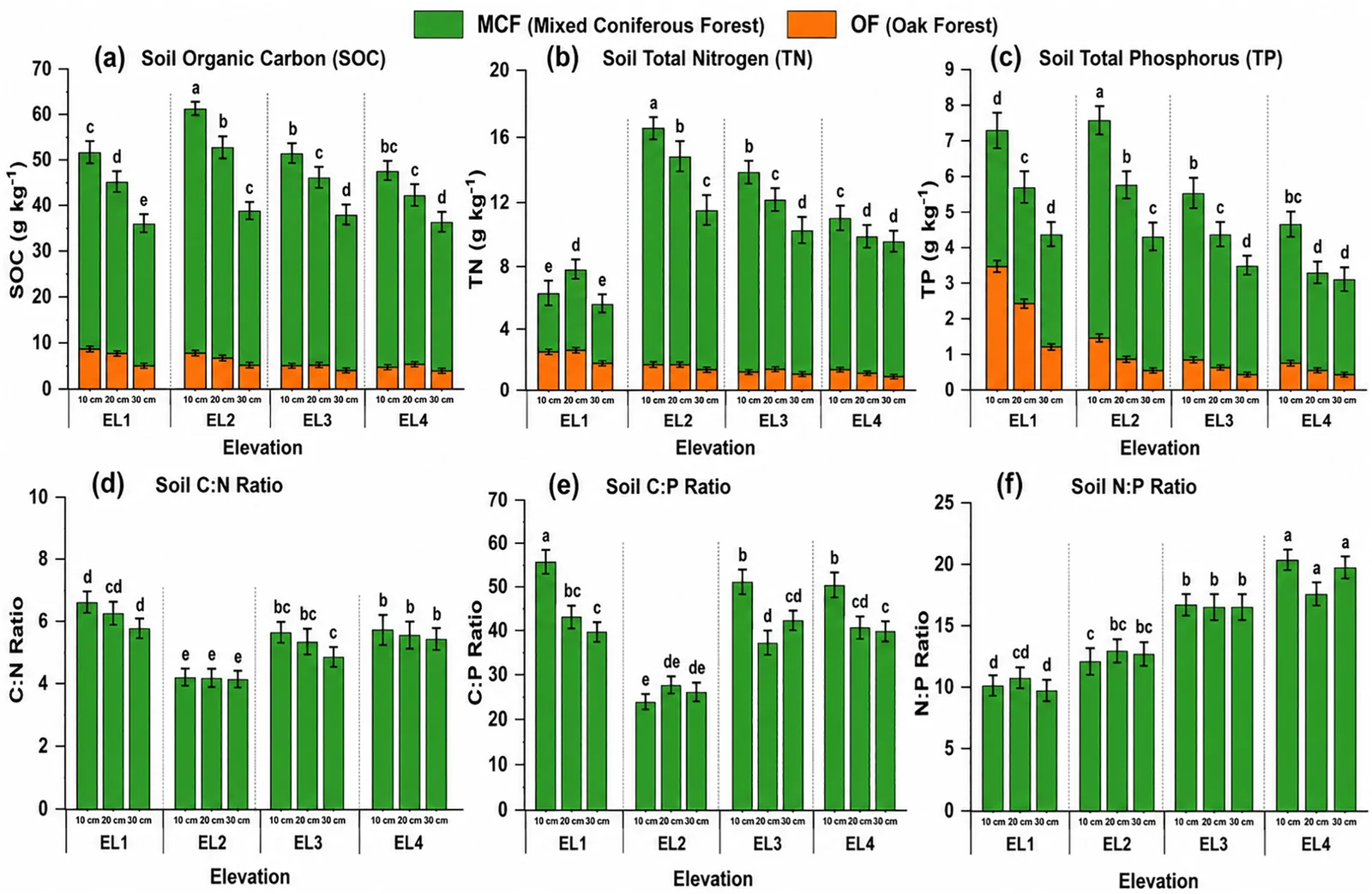
Response of soil C, N, and P contents of Oak Forest (OF) and Mixed Coniferous Forest (MCF) in the different depths of soil at three different clusters (locations) on four different elevations. Different letters indicate significant differences among different altitudes for the same index at the 0.05 level (p < 0.05).

However, soil N content was significantly improved at a 10 cm depth of soil for both MCF and OK forests under different elevations Fig 1. Results showed that soil total N content was significantly improved in the MCF compared with that of the Oak forest under 20 cm depth of soil at elevation EL-2. The Oak Forest reported higher soil total N contents, such as 3.8 mg kg^−1^, 3.3 mg kg^−1^, 2.7 mg kg^−1^, and 2.3 mg kg^−1^ at EL-2, followed by EL-3, EL-4, and EL-1 under a 10 cm depth of soil. Similar trends were noted in the MCFwhere nitrogen content was significantly improved by 16.8 mg kg^−1^ (EL-2), 15.1 mg kg^−1^ (EL-3), 13.1 mg kg^−1^ (EL-1), and 12.4 mg kg^−1^ (EL-4) at a 10 cm depth of soil. Moreover, the comparison results of soil total N between MCF and OF showed that the maximum soil total N was observed in MCF compared with that of OF.

The significant results reported by MCF and OK forests at different soil depths and different elevations of soil total P are shown in Fig 1. Results showed that soil total P content was significantly improved in the MCF compared with that of the Oak forest under 10 cm depth of soil at elevation EL-2. The Oak Forest reported higher total P contents, such as 3.1 mg kg^−1^, 2.7 mg kg^−1^, 2.2 mg kg^−1^, and 1.7 mg kg^−1^ at EL-2, followed by EL-3, EL-1, and EL-4 under a 10 cm depth of soil. The soil total P content decreased with the increase in depth. A similar trend was noted in the MCF that P content was significantly enhanced by 6.3 mg kg^−1^ (EL-2), 4.2 g kg^−1^ (EL-3), 3.4 g kg^−1^ (EL-1), and 3.1 g kg^−1^ (EL-1) at a 10 cm depth of soil. Moreover, the comparison results of soil total P showed that MCF contained more soil P than OF. Further, the soil CNP ratios documented that soil CN was significantly increased at 10 cm soil depth, and was then gradually decreased with the increase in depth. Moreover, EL-2 showed good results in CN, while EL-1 showed good results for CP and NP ratios. Thus, it is concluded that MCF provides better soil nutrients compared with those of OF.

### 3.2. Changes in plant stoichiometry (MCF and OF)

The statistical analysis of the data revealed significant changes in the CNP and its ratios in the leaf, branches, bark, and litter in the MCF and oF plants at different elevations Table 1. The increase in the carbon, nitrogen, and phosphorus content of the Oak leaf was 458.4 mg kg^−1^, 4.42 mg kg^−1^, and 2.54 mg kg^−1,^ respectively, reported from EL-2, and found that this elevation has more nutrients in the leaf compared with other elevations. Moreover, the C:N ratio in the leaf was significantly improved at EL-2, while CP and NP ratios were found not significant (Table 1). In addition, plant CNP and ratios were increased in the branches (41.1 mg kg^−1^, 2.8 mg kg^−1^, 1.7 mg kg^−1^), bark (32.1 mg kg^−1^, 1.91 mg kg^−1^, and 1.46 mg kg^−1^), and litter (52.5 mg kg^−1^, 6.4 mg kg^−1^, and 0.8 mg kg^−1^) at EL-2.

**Table 1:** Stoichiometric ratio of carbon, nitrogen, and phosphorus in the leaf, branches, bark, and litter of Oak and mixed coniferous forests at different elevations in the north mountain of Pakistan.

	Treatments	C	TN	TP	C:N	C:P	N:P
**Oak**	EL-1	340.7C	3.57B	1.95C	111.4B	450.7	4.05
Leaf	EL-2	458.4A	4.42A	2.54A	121.2A	465.1	3.84
	EL-3	417.1B	4.22A	2.39B	115.3AB	450.7	3.91
	EL-4	364.1C	3.66B	1.92C	116.4AB	490.4	4.22
							
	EL-1	25.9D	2.2C	0.9D	13.8B	76.2AB	5.5A
Branches	EL-2	41.1A	2.8A	1.6A	17.0A	65.2B	3.8B
	EL-3	38.2B	2.7A	1.3B	16.6A	75.7AB	4.6AB
	EL-4	34.1C	2.5B	1.1C	16.1A	81.3A	5.1A
							
	EL-1	17.0D	1.29C	0.72D	15.4B	62.3A	4.06A
Bark	EL-2	32.1A	1.91A	1.46A	19.7A	57.0A	2.92A
	EL-3	29.3B	1.78A	1.13B	19.2A	66.7A	3.48A
	EL-4	25.2C	1.56B	0.91C	18.8A	71.1A	3.81A
							
	EL-1	31.7C	4.5C	0.5C	8.3BC	154.2A	18.7A
Litter	EL-2	52.5A	6.4A	0.8A	9.5A	165.7A	17.4C
	EL-3	39.7B	5.3B	0.6B	8.7B	159.7A	18.3AB
	EL-4	28.2C	4.2C	0.5C	7.8C	137.3B	17.6BC
							
**MCF**							
Leaf	EL-1	346d	11.1c	1.3c	36.4a	694ab	19.1
	EL-2	472a	16.1a	1.9a	34.2ab	631b	18.5
	EL-3	431b	15.2a	1.7b	33.0b	669ab	20.3
	EL-4	382c	12.6b	1.4c	35.3ab	719a	20.4
							
Branches	EL-1	37.7B	4.9C	0.9C	9.0B	110.7 A	12.2B
	EL-2	61.5A	8.1A	1.1A	8.9B	141.1A	16.0A
	EL-3	56.4A	6.1B	1.1AB	10.8A	135.7A	12.6B
	EL-4	43.9B	5.2C	0.9BC	9.9AB	121.3A	12.2B
							
Bark	EL-1	29.6B	2.6C	0.74C	13.5AB	111.2A	8.3B
	EL-2	53.4A	5.8A	0.93A	10.8B	147.5A	13.8A
	EL-3	48.3A	3.8B	0.88AB	14.8A	141.2A	9.6B
	EL-4	35.8B	2.9C	0.69BC	14.6A	124.3A	8.5B
							
Litter	EL-1	40.5D	5.8C	0.3C	8.2B	312.3A	38.6A
	EL-2	72.8A	9.0A	0.6A	9.4AB	325.8A	34.7A
	EL-3	63.7B	7.0B	0.5AB	10.6A	310.2A	29.2A
	EL-4	51.9C	6.1C	0.4BC	9.9A	341.7A	34.3A
							
Oak MCF		493.8B	13.3B	5.1A	158.7A	757.3B	30.3B
		556.6A	30.6A	3.9B	67.3B	1259.1A	77.1A

Note: Different capital letters indicate significant differences for leaves, branches, and litter among different altitudes, respectively (p < 0.05).

MCF plant Stoichiometry showed significant changes among different parts of the plant at different elevations in Table 1. Plant carbon, nitrogen, and phosphorus content of the MCF leaf was increased significantly by 472 mg kg^−1^, 16.1 mg kg^−1^, and 1.9 mg kg^−1^ at EL-2, and found that this elevation has more nutrients in the leaf compared with other elevations. Moreover, the C:N ratio in the leaf was significantly improved at EL-2, while CP and NP ratios were found not significant (Table 1). In addition, plant CNP and ratios were increased in the branches (61.5 mg kg^−1^, 8.1 mg kg^−1^, 1.1 mg kg^−1^), bark (53.4 mg kg^−1^, 5.8 mg kg^−1^, and 0.93 mg kg^−1^), and litter (72.8 mg kg^−1^, 9.0 mg kg^−1^, and 0.6 mg kg^−1^) at EL-2.

### 3.3. Response of elevations on soil and plant heavy metals

The results of soil heavy metals of MCF and OF forests at different soil depths for different elevations are presented in Fig 2. Results showed that soil Ni, Pb, Cd, Cu, and Fe were significantly decreased in the MCF compared with those of the Oak forest under a 10 cm of soil at elevation EL-2. The Oak Forest reported higher Ni, Pb, Cd, Cu, and Fe at different depths of soil at EL-1, followed by EL-4 and EL-3. Similar trends were noted in the MCF. Moreover, soil Zn was significantly improved in MCF and OF plants at EL-2 elevation compared with that of other elevations.

**Fig 2:**
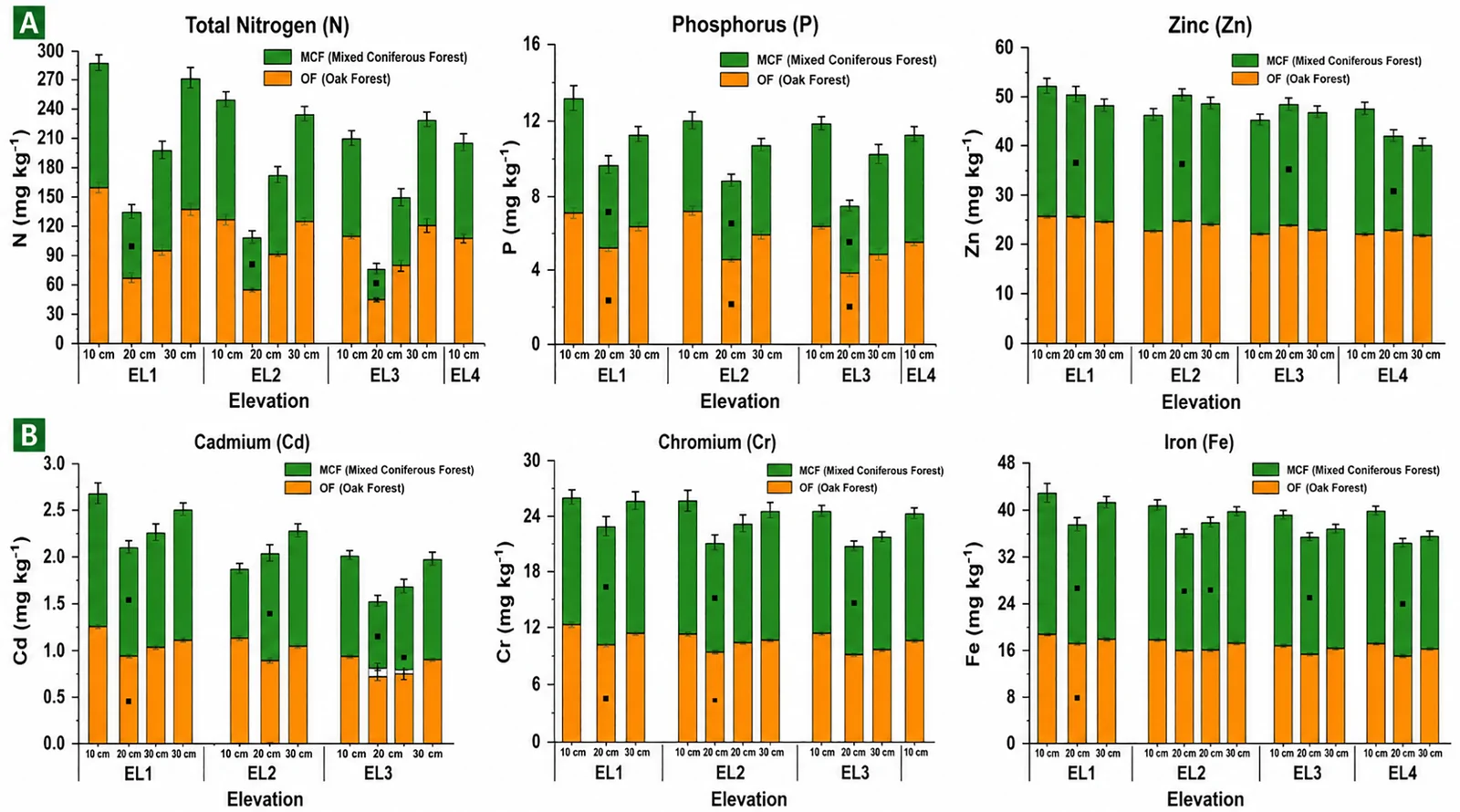
Soil heavy metal concentrations (Ni, Pb, Zn, Cd, Cu, and Fe) across the four elevation classes (EL-1, EL-2, EL-3, and EL-4) in mixed coniferous forest (MCF) and oak forest (OF). Error bars represent standard error (SE).

The statistical analysis of the data revealed significant changes in the heavy metal contents in the leaf, branches, bark, and litter in the MCF and OF plants at different elevations Table 2. The increased in the Ni, Pb, Zn, Cd, Cu, Fe contents of the Oak leaf was 11.1 mg kg^−1^, 12.9 mg kg^−1^, and 18.9 mg kg^−1^, 0.75 mg kg^−1^, 14.1 mg kg^−1^, 563 mg kg^−1^ reported from EL-1, and found that this elevation has maximum heavy metals contents in the leaf compared with others elevations, followed by EL-4, EL-3, and EL-2. In addition, MCF plant heavy metals were increased in the branches (8.97 mg kg^−1^, 8.98 mg kg^−1^, 18.5 mg kg^−1^, 0.80 mg kg^−1^, 13.4 mg kg^−1^, and 554 mg kg^−1^), bark (7.89 mg kg^−1^, 8.46 mg kg^−1^, 17.4 mg kg^−1^, 0.62 mg kg^−1^, 11.4 mg kg^−1^, and 517 mg kg^−1^), and litter (5.91 mg kg^−1^, 6.42 mg kg^−1^, 12.5 mg kg^−1^, 0.53 mg kg^−1^, 9.02 mg kg^−1^, 463 mg kg^−1^) at EL-1 (Table 2). Further, MCF plant showed increasing trend in the contents of Ni, Pb, Zn, Cd, Cu, and Fe compared with that of OF plants such as MCF (26.6 mg kg^−1^, 29.29 mg kg^−1^, 50.09 mg kg^−1^, 2.25 mg kg^−1^, 38.59 mg kg^−1^, and 1819 mg kg^−1^), and in OF plants (23.4 mg kg^−1^, 28.0 mg kg^−1^, 39.4 mg kg^−1^, 1.78 mg kg^−1^, 29.7 mg kg^−1^, 1625 mg kg^−1^).

**Table 2:** The concentration of heavy metals (mg kg^-1^) in the leaf, branches, bark, and litter of Oak and mixed coniferous forests at different elevations in the north mountain of Pakistan.

	Treatments	Ni	Pb	Zn	Cd	Cu	Fe
**Oak**	EL-1	11.1A	12.9A	18.9A	0.75A	14.1A	563A
Leaf	EL-2	6.6C	7.3C	10.7C	0.41C	8.2D	407C
	EL-3	8.0B	10.3B	12.0C	0.59B	10.8C	466B
	EL-4	10.3A	12.6A	15.9B	0.71A	13.2B	526A
							
	EL-1	8.21A	9.47A	15.3A	0.62A	9.30A	425AB
Branches	EL-2	3.68C	5.93B	7.93B	0.28C	6.50C	383B
	EL-3	5.11B	7.87SB	10.2B	0.48B	7.67BC	381B
	EL-4	7.38A	9.17A	13.0A	0.61A	8.80AB	458A
							
	EL-1	7.01A	6.90A	12.1A	0.50A	6.60A	390AB
Bark	EL-2	2.48C	3.36B	4.73B	0.16C	4.33C	348B
	EL-3	3.91B	5.30AB	7.03B	0.36B	5.37B	347B
	EL-4	6.18A	6.60A	9.77A	0.49A	6.27A	423A
							
	EL-1	4.71A	4.92A	8.67A	0.39A	5.40A	358AB
Litter	EL-2	1.78C	1.38B	1.33B	0.12C	3.13C	316B
	EL-3	3.21B	3.32AB	3.63B	0.26B	4.17B	315B
	EL-4	4.08AB	4.62A	6.37A	0.37A	5.07A	391A
							
**MCF**							
Leaf	EL-1	12.3A	13.9A	19.8A	0.84A	14.8A	576A
	EL-2	7.78C	8.34C	11.6C	0.50C	8.94D	420C
	EL-3	9.21B	11.3B	12.9C	0.68B	11.5C	479B
	EL-4	11.5A	13.6A	16.8B	0.80A	13.9B	539A
							
Branches	EL-1	8.97A	8.98A	18.5A	0.80A	13.4A	554A
	EL-2	4.44C	3.44C	10.2C	0.46C	7.60D	398C
	EL-3	5.87B	6.38B	11.6C	0.64B	10.1C	457B
	EL-4	8.14A	8.68A	15.5B	0.76A	12.6B	517A
							
Bark	EL-1	7.89A	8.46A	17.4A	0.62A	11.4A	517A
	EL-2	3.36C	2.92C	9.16C	0.28C	5.52D	361C
	EL-3	4.79B	5.86B	10.5C	0.46B	8.06C	420B
	EL-4	7.06A	8.16A	14.4B	0.58A	10.5B	480A
							
Litter	EL-1	5.91A	6.42A	12.5A	0.53A	9.02A	463A
	EL-2	1.38C	0.88C	4.26C	0.19C	3.18D	307C
	EL-3	2.81B	3.82B	5.59C	0.37B	5.72C	366B
	EL-4	5.08A	6.12A	9.52B	0.49A	8.15B	426A
							
Oak MCF		23.4B	28.0AB	39.4B	1.78AB	29.7B	1625B
		26.6A	29.29A	50.09A	2.25A	38.59A	1819A

### 3.4. Relationship among soil and plant nutrients, and heavy metals of MCF and OF plants

The correlation analysis among soil and plant stoichiometry, heavy metals was presented in Fig 3 (A and B), which documented that soil and plant stoichiometry were significantly correlated with each other. Further, strong positive correlations were noted among soil CNP and plant CNP of the MCF plant (Fig 3A), while negative correlations were noted with soil and plant heavy metals, and soil nutrients with plant heavy metals. Positive relationships were noted among plant heavy metals and among soil heavy metals of MCF plants. Except for soil Zn, all soil heavy metals were correlated positively with all plant heavy metals. Moreover, correlation analysis among soil and Oak plant nutrients and heavy metals showed a highly significant correlation compared with that of MCF. Soil CNP has a strong correlation with plant CNP, and soil CN, NP, Ni, Pb, CD, Cu, and Fe have a strong correlation with all plant heavy metals (Fig 3B).

**Fig 3:**
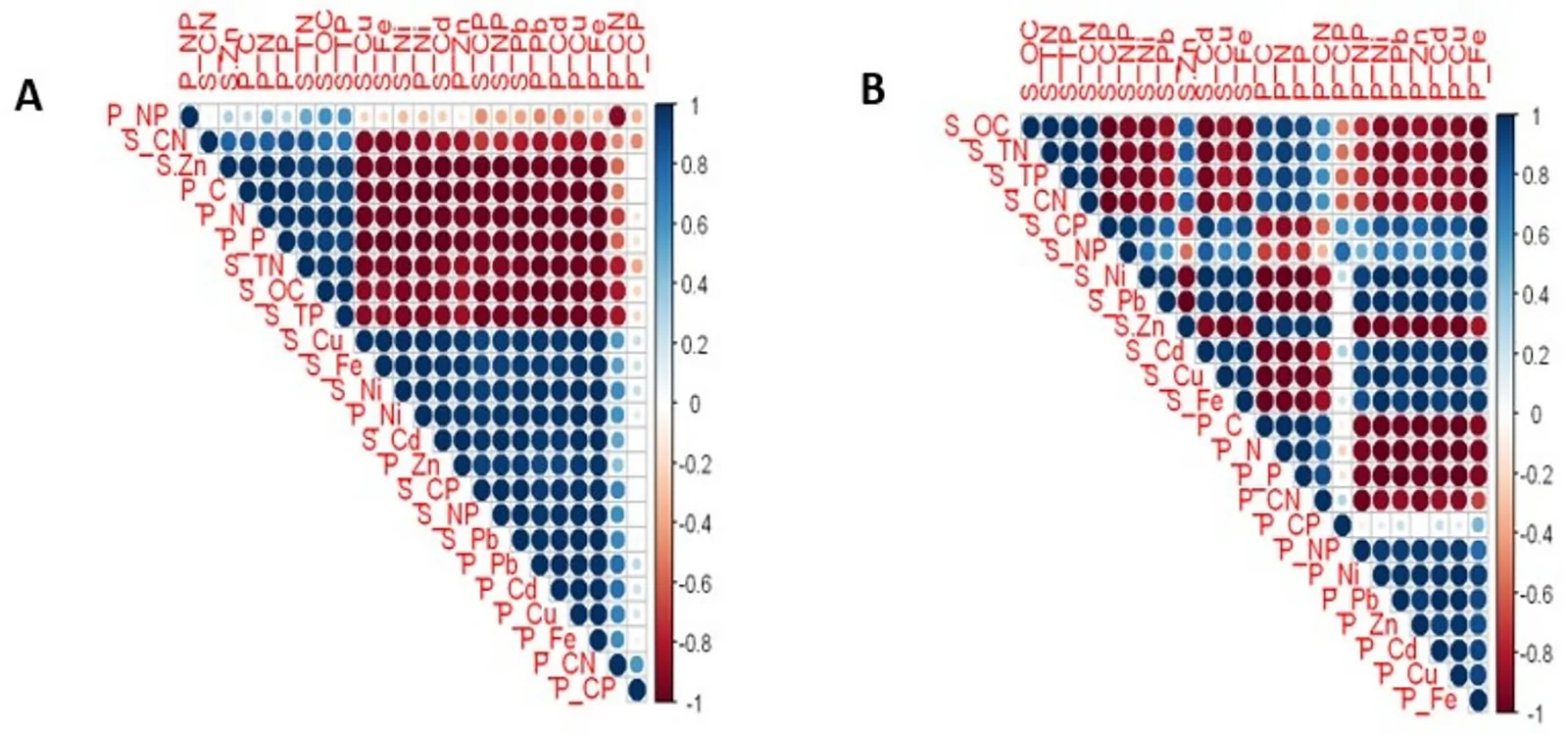
Correlation analysis among soil and plant nutrients, heavy metals of MCF (A), and OF (B) forest ecosystem.

In addition, the regression analysis explained the role of elevations for soil and plant stoichiometry, and heavy metals presented in Fig 4A and B. The soil stoichiometry was significantly related to plant stoichiometry, and showed that all soil variables significantly affected and presented a linear trend with plant CNP in MCF (Fig 4A), while soil N and soil P showed no significant relations with plant CNP traits; however, soil C showed a significant linear trend with plant CNP traits in Oak Forest plants (Fig 4B). Moreover, Fig 5A, B, showed the principal components analysis and explained the variation on both axises (PC1, 10.8%, and PC2, 74.2%) for MCF plants, and (PC1, 18.3%, and PC2, 66.4%) for OF plants, and reported that most of the soil and plant stoichiometry were lies at EL-2 followed by EL-3 elevations, and thus it is concluded that EL-2 is the best elevation, which showed improvements in soil and plant stoichiometry, and decline of heavy metals for both MCF and OF plants (Fig 6A, B).

**Fig 4:**
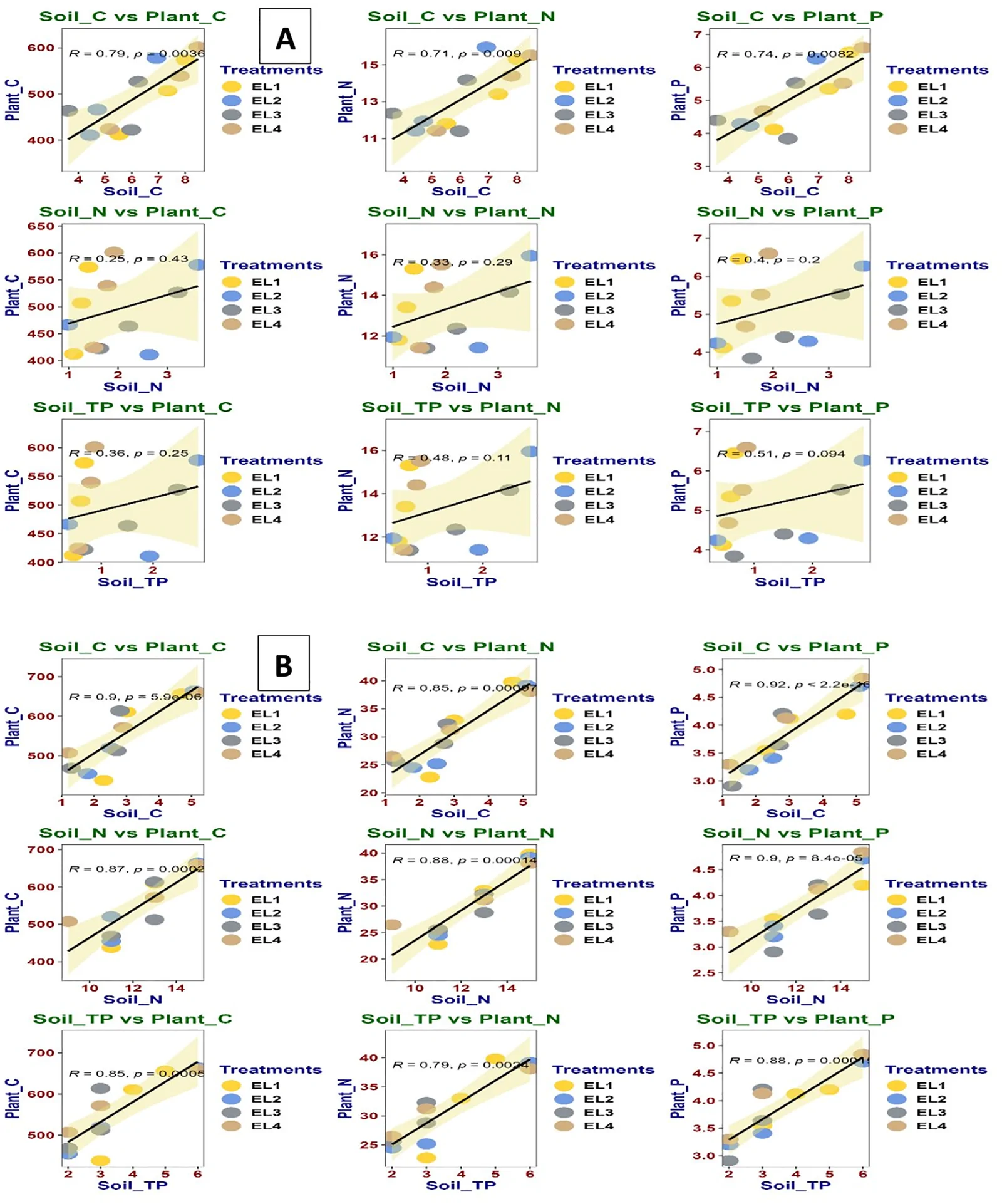
Altitude patterns and soil C, N, and P relationships with the C, N, and P of. MCF (A), OF ecosystem (B).

**Fig 5:**
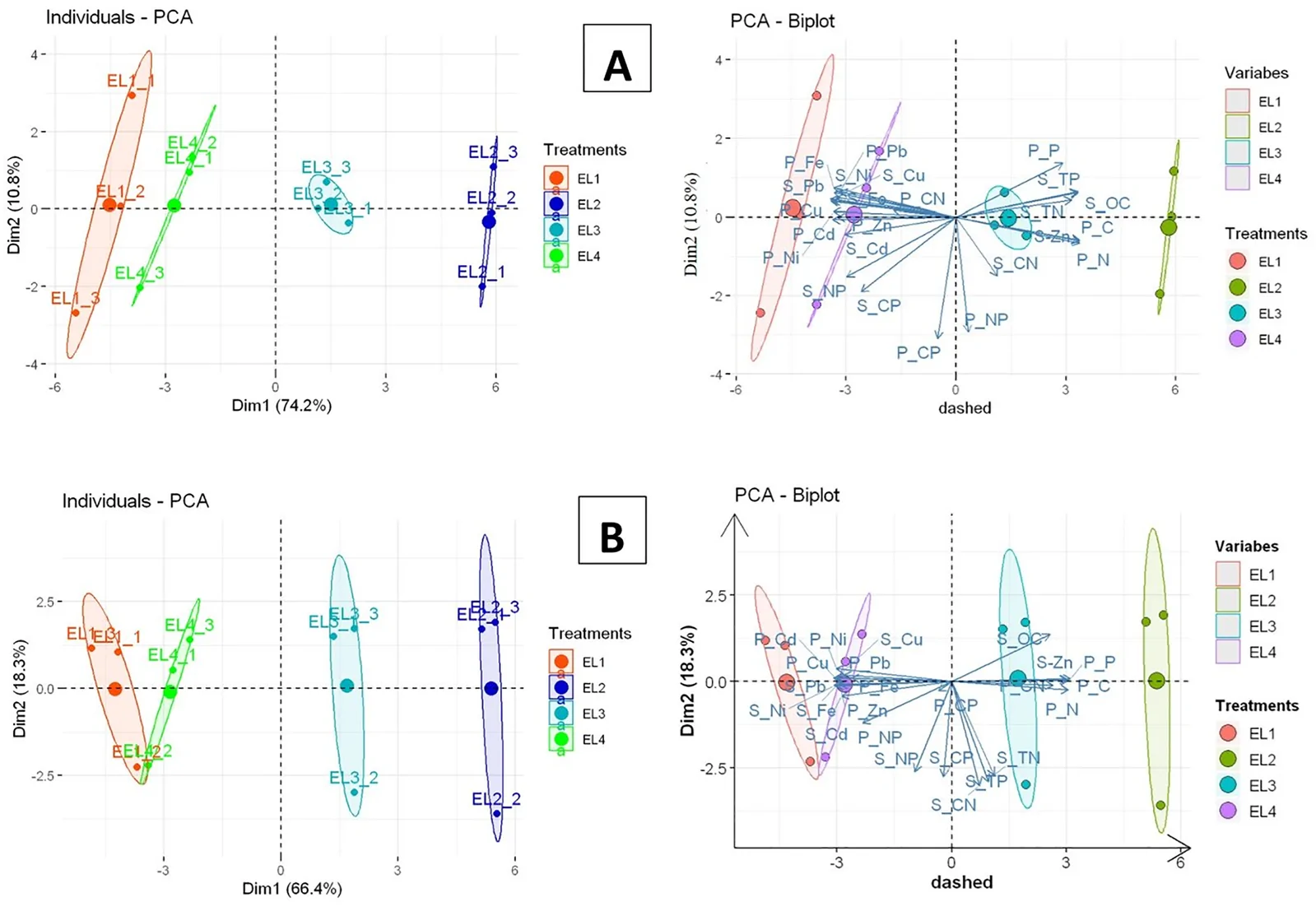
Principal components analysis showed the comparison among different elevational gradients with soil and plant nutrients, and heavy metals (A: MCF, B: OF).

**Fig 6:**
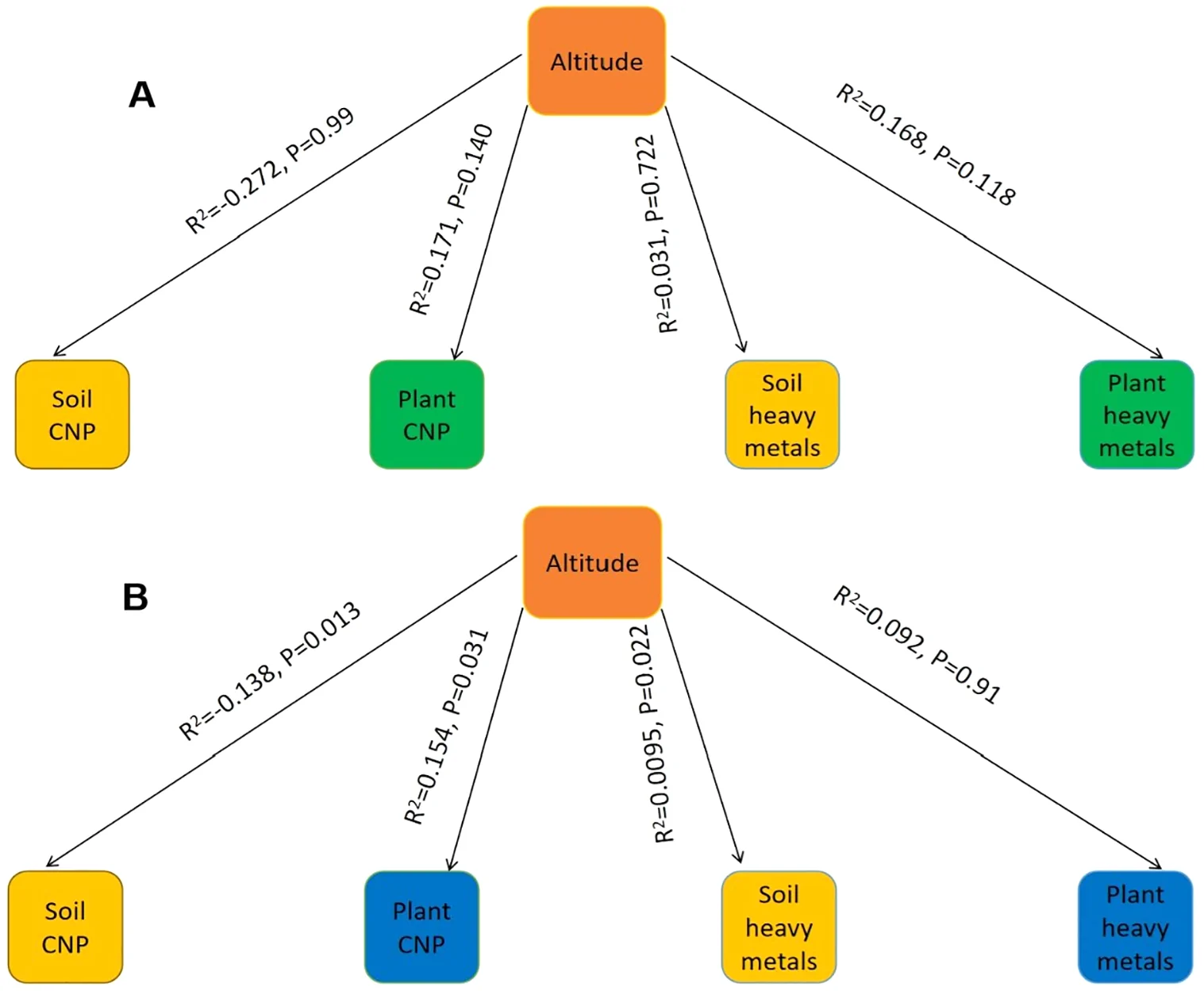
Structural equation models (SEM) of altitude on soil and plant nutrients, heavy metals distributions. Numbers adjacent to arrows are the standardized path coefficient (equivalent to correlation coefficients). A: OF, B: MCF.

## 4. Discussion

The stoichiometry of soil and plant explore the the proportion of necessary elements such as carbon (C), nitrogen (N), and phosphorus (P) between soil and plants, and thus explain how the analyze how the stoichiometry of C, N, and P in soil influences nutrient uptake and plant composition [38], and fundamentally highlight the availability of plants and soil nutrients within forest ecosystems [2]. Therefore, soil–plant stoichiometry provides an effective framework for understanding nutrient limitation, ecosystem productivity, and the ecological functioning of mountain forest ecosystems.

This experiment explores that at elevation (EL-1), soil C, N, and P declined, while an improvement was noted at elevation (EL-2). Further, the decline in the soil C, N, and P contents was noted at the upper elevations (EL-3 and EL-4). This data was confirmed with the results that soil stoichiometric characteristics first declined and then enhanced with the increase in the elevations [39]. The higher nutrient concentrations observed at EL-2 suggest that intermediate elevations provide more favorable environmental conditions for nutrient accumulation than either lower or higher elevations.

Our results showed that the C, N, and P contents in the leaf, branches, bark, and litter were enhanced at elevation (EL-2), and then trended to decline at higher elevations. This trend closely followed the variation in soil nutrient availability, indicating a strong coupling between soil nutrient status and plant nutrient accumulation., The decrease in the nutrients of soil is likely associated with this evidence and strengthens the result to the severe environmental stress at upper elevations [40]. Furthermore, at high elevations, due to low temperature, nutrient deficiencies become more pronounced, which contributes to reductions in soil and plant nutrients, supporting the patterns observed in the present study [41]. Furthermore, soil nutrient contents were influenced by the uptake of nutrients and thus affected soil ecology [42].

Moreover, this work revealed that the contents of nutrients in the fresh leaf were more than those in the soil, which indicates a slow turnover of nutrients from the fresh leaf to the soil. This finding suggests that nutrient recycling through litter decomposition is relatively slow, particularly under cooler environmental conditions. This determination suggests that the lower decomposition rate at higher altitudes decreases the nutrient flow process. It is concluded that our phenomenon attests that the different conditions of the climate instantly influenced the status of the nutrients of the forest ecosystems [43]. The observed pattern highlights the important role of elevation-driven climatic variation in regulating nutrient cycling and ecosystem functioning.

Our results determined that at the initial soil depth (0–10 cm), the ratios of C:N, C:P, and N:P declined, possibly because the decomposition rate of soil organic matter was supported on the C:N and the decomposition rate has been enhanced due to the decrease in the C:N [44]. Nevertheless, the improvement in the ratios of CNP was observed with the increase in the depth at different elevations. These changes in stoichiometric ratios indicate that nutrient availability and microbial decomposition processes vary considerably with soil depth and elevation, reflecting differences in organic matter turnover and nutrient mineralization.

Furthermore, our findings reported that the leaf, branches, bark, and litter of Oak and mixed coniferous plants significantly enhanced the content of carbon at different elevations, with the highest values recorded at elevation (EL-2), followed by EL-3. This report is consistent with previous studies showing that carbon content is generally higher in green broadleaf forests [1]. Also, the decline in the content of C was noted with the increase in the forest elevations. A likely explanation is that lower temperatures at higher elevations reduce photosynthetic activity, microbial decomposition, and nutrient mineralization, thereby limiting carbon accumulation in plant tissues. The unstable effect was accordingly due to the decline in the temperature at high altitudes, which limits the response of microbes, and has a poor return of the C ecosystem. Therefore, at high altitudes, lower carbon concentrations in leaves, branches, and litter have been observed [45]. Collectively, these findings demonstrate that elevation not only regulates soil nutrient availability but also influences plant nutrient allocation and carbon sequestration through its effects on climate and biological activity.

The process by which the resorption of nutrients in plants reallocates nutrients from senescent tissues actively growing or storage structures is an important adaptive mechanism that enhances nutrient-use efficiency under nutrient-limited conditions. This chemical change refers to a biological or biochemical process, such as microbial activity, enzymatic transformation, or internal plant signaling, that helps retain vital nutrients like nitrogen (N), phosphorus (P), and potassium (K) within the plant-soil system. Instead of relying heavily on continuous external nutrient inputs (e.g., fertilizers), plants benefit from improved internal recycling and conservation of these nutrients [46]. Such efficient nutrient resorption is particularly important in mountain ecosystems, where nutrient availability decreases with increasing elevation and plants must maximize nutrient conservation to sustain growth.

By increasing the efficiency of nutrient recycling, plants are able to reuse nutrients that would otherwise be lost through leaching, volatilization, or immobilization in unavailable forms. For example, organic matter decomposition or root exudate-driven microbial activity can release bound nutrients, making them available again for plant uptake [47]. These belowground processes strengthen the linkage between soil nutrient availability and plant nutrient acquisition, thereby maintaining ecosystem productivity despite environmental constraints.

To overcome environmental challenges require sufficient nutrients to maintain physiological processes and enhance stress tolerance [48]. The environmental issues affect plant reproduction and thus influence the decomposition of the litter, which releases nutrients and balance the nutrients cycling’s [49]. Therefore, efficient nutrient recycling through litter decomposition becomes increasingly important under stressful environmental conditions associated with higher elevations.

Nevertheless, limited information is still available, although how various nutrients move during the resorption process. Furthermore, plant species growing under similar environmental conditions may exhibit convergent adaptations in functional traits, physiology, and genetics [50]. Such adaptive responses enable plants to optimize nutrient acquisition and conservation while maintaining ecosystem stability under changing environmental conditions. This effect will assist ecologists with improving their biogeochemical models, and increase their knowledge of nutrient cycling. Overall, these findings emphasize that nutrient resorption and internal nutrient recycling are fundamental mechanisms regulating soil–plant stoichiometry along elevational gradients.

The correlation analysis revealed that soil nutrients were strongly related to plant nutrients and showed a linear trend, which declined with the upper elevations. This strong relationship demonstrates the close coupling between belowground nutrient availability and aboveground plant nutrient composition These results were confirmed that due to the strong relations between environment, i.e., due to the increase in the elevation the temperature was decline and humidity increased, and hence reduced the microbial activity and finally decreased the soil organic matter decomposition and alleviated the accumulation of C and N [51]. These findings suggest that climatic factors associated with elevation indirectly regulate plant nutrient status by influencing microbial-mediated nutrient cycling.

Various findings showed that soil nutrients were influenced by the site or locations, such as altitude and latitude [52]. Therefore, in this work elevation was identified as one of the major environmental factors controlling soil nutrient dynamics. The contents of soil C, N, and P were correlated significantly with respect to elevations (Fig 4). This relationship further indicates that elevation influences nutrient availability through changes in temperature, moisture conditions, and biological activity.

Our results were aligned with the findings from the previous work that the soil C and N contents showed a significant linear trend with the elevation [53]. Further, it was noted that the soil N and P contents were improved with the elevations [54]. The consistency between our findings and previous studies strengthens the evidence that elevational gradients are key drivers of nutrient stoichiometry in forest ecosystems. Moreover, the observed relationships suggest that changes in nutrient availability may directly influence vegetation productivity and ecosystem resilience under changing climatic conditions.

Our experimental results analyzed the response of different elevations to soil heavy metals. Results documented a significant variation in heavy metal levels with increasing elevation, which can be attributed to a sequence of natural and environmental factors. These findings indicate that elevation acts as an important regulator of heavy metal distribution by influencing both natural geochemical processes and ecosystem characteristics. Firstly, the observed trend of elevated heavy metal concentrations at higher elevations suggests a potential influence of geological and mineralogical characteristics inherent to mountainous terrains. Elevated areas often contain mineral deposits rich in metals such as lead, zinc, and cadmium, which can naturally contribute to soil contamination [7]. Moreover, the weathering of rocks and soil erosion processes at higher altitudes may facilitate the mobilization and accumulation of these metals in the soil profile [55]. Therefore, variations in parent material and weathering intensity likely contribute to the spatial heterogeneity of heavy metal concentrations observed across the elevation gradient.

Further, our results declared that at lower elevation the heavy metal concentrations were higher and then declinedat EL-2, while an increase in the metals were noted with the increase in the elevations. This nonlinear trend suggests that heavy metal accumulation is controlled by multiple interacting environmental processes rather than elevation alone. This may be due to the fact that lower elevations tend to accumulate higher concentrations of soil heavy metals due to several factors, including natural geological processes, runoff from higher elevations, and anthropogenic activities. Natural processes like weathering and erosion transport heavy metals from bedrock to lower areas, while runoff from higher areas can carry contaminants and settle in lower basins. Additionally, areas with lower elevations often experience more intensive human activities, such as industrialization and agriculture, which can further contribute to heavy metal pollution. The comparatively lower heavy metal concentrations recorded at EL-2 may therefore reflect reduced anthropogenic influence together with more balanced ecological conditions that favour nutrient cycling while limiting metal accumulation.

Moreover, the impact of atmospheric deposition cannot be overlooked. The upper elevations are more exposed to long-range atmospheric transportation of pollutants, including heavy metals rise from originating from industrial emissions and vehicular emissions in urbanized regions [56]. These atmospheric compounds can adhere to the surface of soil and therefore enhance heavy metal accumulation in mountain soils [57]. Forest canopies at higher elevations can further intercept airborne particles through canopy surfaces, litterfall, and throughfall, thereby enhancing the deposition and retention of heavy metals within forest soils. Consequently, even relatively undisturbed mountain ecosystems may accumulate measurable concentrations of atmospheric contaminants over long periods.

These interpretations are consistent with the statistical results, which reveal strong correlations between elevation and certain heavy metal content. In particular, cadmium and lead were the metals with the strongest associations, highlighting their potential as markers of elevation-related environmental contamination. The strong relationships observed for cadmium and lead further suggest that these metals are more responsive to environmental changes along elevational gradients than other elements and may therefore serve as useful indicators for monitoring ecological health in mountain forest ecosystems. Their accumulation may also influence nutrient cycling through interactions with soil microorganisms and plant nutrient uptake, emphasizing the close relationship between heavy metal dynamics and soil–plant stoichiometry.

Overall, the combined analysis of nutrient stoichiometry and heavy metal dynamics demonstrates that elevation simultaneously regulates nutrient availability and contaminant distribution in mountain forests. The interaction between climatic conditions, soil properties, vegetation characteristics, and geochemical processes determines both ecosystem nutrient status and heavy metal accumulation. These findings provide valuable baseline information for understanding ecosystem functioning in the forests of the Hindu Kush–Karakoram–Himalayan region and support the development of sustainable forest management strategies aimed at mitigating heavy metal pollution while maintaining ecosystem productivity under changing environmental conditions.

## 5. Conclusions

This study demonstrates that elevation influences soil–plant stoichiometry and heavy metal distribution in mixed coniferous and oak forests of northern Pakistan. Across the four elevation classes, EL-2 generally exhibited the highest concentrations of soil and plant C, N, and P, whereas nutrient concentrations tended to decline at higher elevations. Heavy metal concentrations also varied with elevation and forest type, with lower soil concentrations of several metals at EL-2 and greater metal accumulation in plant tissues at EL-1. The observed relationships between soil and plant nutrients and heavy metals highlight the importance of elevation and forest type in regulating nutrient cycling and metal dynamics. These findings provide a useful baseline for ecological monitoring and sustainable management of mountain forest ecosystems in Gilgit-Baltistan.

## Supporting information

S1 Data All relevant Background Data_R1. (XLSX)

S1 Fig Supplemental Figure 1. (BMP)
